# Molecular detection of hepatitis E virus in sheep from southern Xinjiang, China

**DOI:** 10.1007/s11262-015-1194-9

**Published:** 2015-04-02

**Authors:** Junyuan Wu, Fusheng Si, Chunyu Jiang, Tao Li, Meilin Jin

**Affiliations:** State Key Laboratory of Agricultural Microbiology, College of Veterinary Medicine, Huazhong Agricultural University, Wuhan, 430070 China; College of Animal Science, Tarim University, Xinjiang, 843300 China; Institute of Animal Science and Veterinary Medicine, Shanghai Academy of Agricultural Sciences, Shanghai, 201106 China

**Keywords:** Hepatitis E virus, Sheep liver, Genotype, ORF2, Phylogenetic analysis

## Abstract

Hepatitis E virus (HEV) is a causative agent of infectious hepatitis in animals and humans both in developing and developed countries. Here, we collected 500 sheep sera and 75 raw sheep liver samples from a slaughterhouse in the southern part of the Xinjiang region, China, along with 26 sera of butchers from the same slaughterhouse. All serum samples were tested for anti-HEV antibody by enzyme-linked immunosorbent assay. Both serum and liver samples were evaluated for the presence of HEV RNA by nested polymerase chain reaction targeting partial nucleotide sequences of open reading frame 2 (ORF2). The results indicate that sheep seroprevalence was 35.20 % (176/500) and that four of the 75 (5.3 %) sheep livers showed detectable amounts of HEV RNA. The seroprevalence of the butchers was 57.7 % (15/26). The four amplicons shared 97.8–100 % nucleotide sequence identity and had pairwise sequence identities of 81.6–85.3 %, 84.2–85.3 %, 82.1–85.3 % and 84.7–97.9 % with the corresponding regions of genotypes 1, 2, 3 and 4 of HEV, respectively. A phylogenetic tree was constructed based on alignments of an amplified 186-bp ORF2 sequence and corresponding reference strains. The analysis showed that the four sheep strains detected in our study formed a lineage within a genotype 4 cluster that contains hb-3, bjsw1, T1, swCH189 and swCH25, all of which belong to genotype 4, subtype 4d. The results indicated a high level of seroconversion in sheep and suggested that sheep liver may be a source of foodborne HEV infection in humans.

## Introduction

Infections with hepatitis E virus (HEV) are an important public health concern in many developing countries in Asia and Africa where sanitation conditions are suboptimal [1–3]. Sporadic cases of HEV infection have also been reported in many industrialised countries where transmission of the virus is mainly zoonotic [4–7]. HEV infections are typically self-limiting and asymptomatic. Nevertheless, symptomatic infections can occur and are often mistaken for drug-induced liver injury [8, 9]. In pregnant women living in countries where HEV is highly endemic, HEV infections have a 20 % mortality rate. Moreover, up to 70 % of people with underlying chronic liver disease may develop an often fatal, fulminant hepatitis [4, 10–13].

HEV is the only species of the genus *Hepevirus*, which belongs to the *Hepeviridae* family [14]. It is a non-enveloped, single-stranded, positive-strand RNA virus with a 7.2-kb genome that consists of three discontinuous open reading frames (ORFs; ORF1, ORF2 and ORF3) and flanking untranslated regions. ORF1 encodes non-structural proteins that are involved in viral replication and viral protein processing. Their putative functional domains may have methyltransferase, papain-like cysteine protease, helicase and RNA-dependent RNA polymerase activities. Other domains are homologous to domains found in other positive-strand RNA viruses, such as Y, X or macro domains and polyproline regions. ORF2 occupies the 3′-terminal part of the genome and encodes a glycoprotein of the viral capsid. ORF3 encodes a cytoskeleton-associated phosphoprotein that is required for virion release from cells and is associated with numerous cellular pathways [15–24].

Although all HEV strains may belong to a single serotype [25, 26], phylogenetic analysis of HEV sequences has led to the recognition of the existence of at least four major genotypes [27–29]. Genotype 1 and 2 HEVs are restricted to humans and are often associated with large outbreaks and epidemics in developing countries in Asia and Africa. Genotype 3 and 4 HEVs have been isolated from humans and from several animal species, including pigs, wild boars, deer and mongooses. Genotype 3 is distributed globally and found equally in industrialised and developing countries, whereas genotype 4 is mainly found in Asian and European countries [14, 30–42]. Recently, new HEV variants have been identified in rats, wild boars, ferrets, bats, chickens and cutthroat trout [43–50], and a cluster of HEV strains has been identified in farm rabbits in China, the USA and France [51–55]. The rabbit strains of HEV from these countries belong to genotype 3 [29, 37]. Many questions concerning HEV infections, however, remain open. For example, it is unclear whether all HEV genotypes can infect the above-mentioned species and whether newly identified HEV variants can infect humans or only animals.

The consumption of contaminated drinking water is generally considered the primary source of HEV transmission to humans. With the discovery of foodborne transmission after ingesting raw or undercooked meat or inner organs, however, this viewpoint is gradually changing [7, 56–59]. Some recent studies indicate that HEV may spread to humans through the food chain from animal reservoirs, such as swine, deer, or wild boar [60, 61]. Hence, studies on HEV circulation in different species providing integrated farm-to-table risk assessments are urgently needed.

In China, Xinjiang is one of the main areas affected by HEV infection, and two large-scale outbreaks have occurred in its southern region [62]. Sheep are raised abundantly in these areas, and mutton and sheep viscera are commonly eaten by local inhabitants, in particular by Uighurs. Wang and Ma [63] confirmed the existence of genotype 4 HEV in sheep faecal samples in this region, and Wu et al. [64] further reported geographical, breed and age-related differences in HEV prevalence. Studies on whether HEV can also be disseminated via sheep liver, however, are limited. The aim of the present study was to acquire serological evidence for HEV infections and to assess the potential for HEV infections to be foodborne in this region.

## Materials and methods

### Serum and liver specimens

**Fig. 1:**
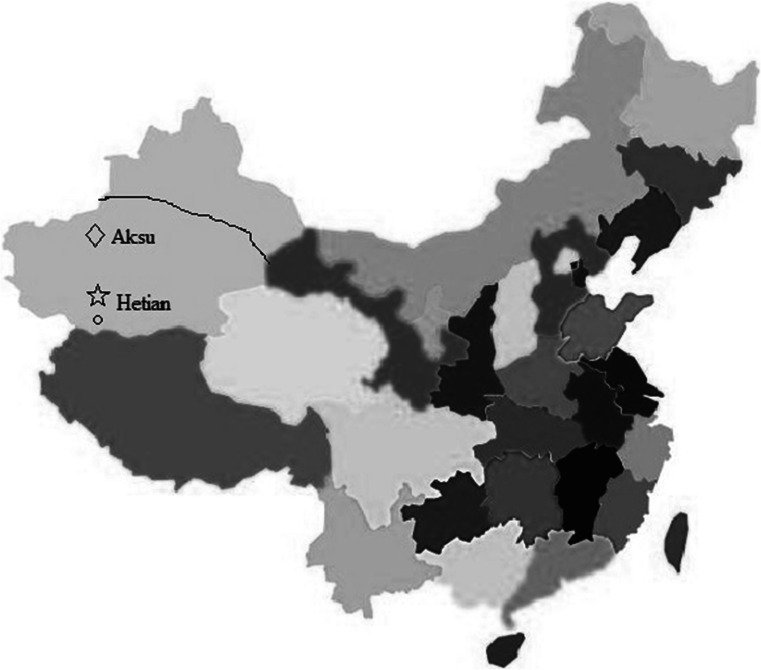
Geographical distribution of the three different farms in Xinjiang. On the map of China, different provinces are marked with *different colours*. The Xinjiang Uighur Autonomous Region is marked in *light blue*. The three farms were distributed in the southern part of Xinjiang, which is demarcated by a *black line*. *Open star* indicates Hetian region. *Open circle* indicates Hetian county of Hetian region where farms A and B were located and which corresponds to the area where the two large-scale outbreaks during 1986 and 1988 occurred. *Open diagonal* indicates Aksu region where farm C was located (Color figure online)

A total of 575 sheep samples from clinically healthy sheep were collected in January and February 2014 at a slaughterhouse in the southern part of Xinjiang region, China (procedures were approved by the Xinjiang Uighur Autonomous Region Animal Care and Use Committee). They included 500 sera (150 of them from sheep <1 year of age, and 350 from sheep of ≥2 years of age) and samples from livers of 75 additional sheep of various ages for which no sera were available. Sera from 26 Uighur butchers working in the same slaughterhouse were also collected, and all of them had worked continuously at the slaughterhouse for more than 3 years (procedures were approved by the Xinjiang Uighur Autonomous Region Centers for Disease Control and Prevention). The sheep came from three different farms from the surrounding area (Fig. 1), i.e., an area where two large-scale outbreaks involving 120,000 infected people had occurred between September 1986 and April 1988. Hetian county is a representative epidemic focus where two human HEV strains were isolated from Uighurs during this time, and in which farms A and B were located [62]. Animal quarantine archives indicated that sheep raised in high density at each farm were gathered from farmers scattered through the region. At the slaughterhouse, the sheep from the three different farms were housed separately for 2 days in sheepfolds before slaughter. Samples were stored at −80 °C until processing.

### Detection of anti-HEV antibody

All serum samples collected from sheep and butchers were tested for anti-HEV antibody using a direct sandwich enzyme-linked immunosorbent assay (ELISA) kit (Cat. No: HEV-0496, Beijing Wantai Biopharmaceutical, Inc.), according to the manufacturer’s instructions.

### RNA extraction and nested reverse transcription polymerase chain reaction (RT-nPCR)

Approximately, 10–20 mg of each liver sample was homogenised on ice with phosphate buffered saline (PBS; 0.01 M, pH 7.2–7.4) using an automatic homogeniser and a sterile micropestle. The tissue suspensions were cleared by centrifugation at 12,000 rpm for 15 min at 4 °C. Duplicate supernatants (150 µl) were prepared and stored at −80 °C. Total RNA was extracted from aliquots of the cleared liver material (or 150 µl serum) with TRIZOL (Invitrogen) according to the manufacturer’s protocol and eluted in 20 µl of RNase-free water.

Extracted viral RNA was used immediately for reverse transcription and cDNA synthesis with a PrimeScript™ II First-strand cDNA Synthesis Kit (Cat. No: 6210A, Takara), followed by nPCR with primers targeting HEV ORF2 regions as described previously. The results of the nPCR strategy for HEV genotyping correlated well with those of phylogenetic analyses conducted on the basis of the complete HEV genome [65]. In our study, first round amplification was achieved with the external forward primers P1 (5′-AATGGWGTTGGYGAGGTC-3′) and the reverse primer P2 (5′-WGARAGCCAAAGCACATC-3′). For nPCR, the internal forward and reverse primers were P3 (5′-TGTTTAATCTTGCTGATACG-3′) and P4 (5′-TGYTGGTTRTCRTAATCCTG-3′), respectively. The two rounds of PCR amplification were both carried out in 25-μl reaction volumes containing 2.5 μl of 10 × PCR buffer, 1 μl of 10 mM dNTPs, 0.5 μl (5 U) Taq DNA polymerase (Roche Molecular Systems), 1 μl (10 μM) each of sense (external or internal) and anti-sense primers, 2 μl template and 17 μl sterile water for 35 cycles of 94 °C for 30 s (additional 5 min for the first cycle), 53 °C for 30 s and 72 °C for 40 s (additional 10 min for the last cycle). Negative (diethyl pyrocarbonate water) and positive (positive swine liver) control samples were included during RNA extraction and first and second PCRs. The reaction system and conditions used in our study were capable of detecting 2.5 ng HEV RNA in liver, which was determined using serial mixtures by adding tenfold dilutions of swine HEV RNA into the swine liver suspensions with the same concentration. To avoid cross-contamination, each phase of the procedure was carried out using various precautions, including the use of separate rooms, specialised equipment, pipettes and disposable gloves. The final PCR product was analysed by 1.2 % (w/v) agarose gel electrophoresis, and the size of the nPCR product was 226 bp, including 40-bp primer regions.

### Sequencing and phylogenetic analysis

Nested PCR products were excised from agarose gels and purified using HiPure Gel Pure DNA Kits (Cat. No: D2110-03, Magen). Purified RT-PCR products were cloned into the pGEM-T Easy Vector (Cat. No: A1380, Promega) and sequenced with an M13 primer. After trimming off the 40-nt primer regions, sequences were assembled and aligned with other swine and human HEV sequences present in the NCBI GenBank. Sequence alignments were generated by CLUSTAL-W (version 2.0). Genetic distances between pairs of viral strains were calculated with MEGA software (version 5.0) using the Kimura two-parameter method. Percent identity was calculated with Lasergene (version 5.03; DNAstar). Phylogenetic trees were constructed by the neighbour-joining method, and 1000 resamplings of the data were used to calculate percentages of the obtained branches. Reference strains for each of the four genotypes, selected to represent the geographical source and host variation, were used for phylogenetic analysis.

### Statistical analysis

To identify a possible correlation between anti-HEV seroprevalence and the age of animals, sheep were divided in two groups (young animals: <1 year old, and adult animals: ≥2 years old). All statistical analyses were performed using SPSS software (version 16.0).

## Results

### Seroprevalence of anti-HEV antibody and HEV RNA

All serum samples were tested for anti-HEV antibody and HEV RNA. The sheep seroprevalence of anti-HEV antibody was 35.2 % (176/500). There were statistically significant differences between the two age groups, with 45.7 % (160/350) positivity among the adult cohort, and 10.7 % (16/150) positivity among young animals (*p* < 0.01). The seroprevalence of butchers was 57.7 % (15/26). None of the serum samples were positive for HEV RNA.

### Detection of HEV RNA in liver samples

To determine whether sheep liver was infected with HEV, 75 raw sheep livers were tested for the presence of HEV RNA by RT-nPCR. At least five samples were harvested and analysed from each liver. Four of the 75 livers gave at least one sample with detectable amounts of HEV RNA (Table 1).

**Table 1:** Number of livers tested for HEV by RT-PCR

Source of liver specimens^a^	No. of livers	No. of positive specimens (%)^b^	Name of HEV strains	Accession numbers
A	30	1(3.3)	XJNJSLhev25	KP294330
B	35	2(5.7)	XJNJSLhev28 XJNJSLhev30	KP294332 KP294333
C	10	1(10)	XJNJSLhev27	KP294331
Total	75	4(5.3)	**/**	

^a^Letters refer to three different farms where the sheep were bred

^b^Numbers in brackets represent the respective percentages

### Phylogenetic analysis of HEV isolated from sheep

**Fig. 2:**
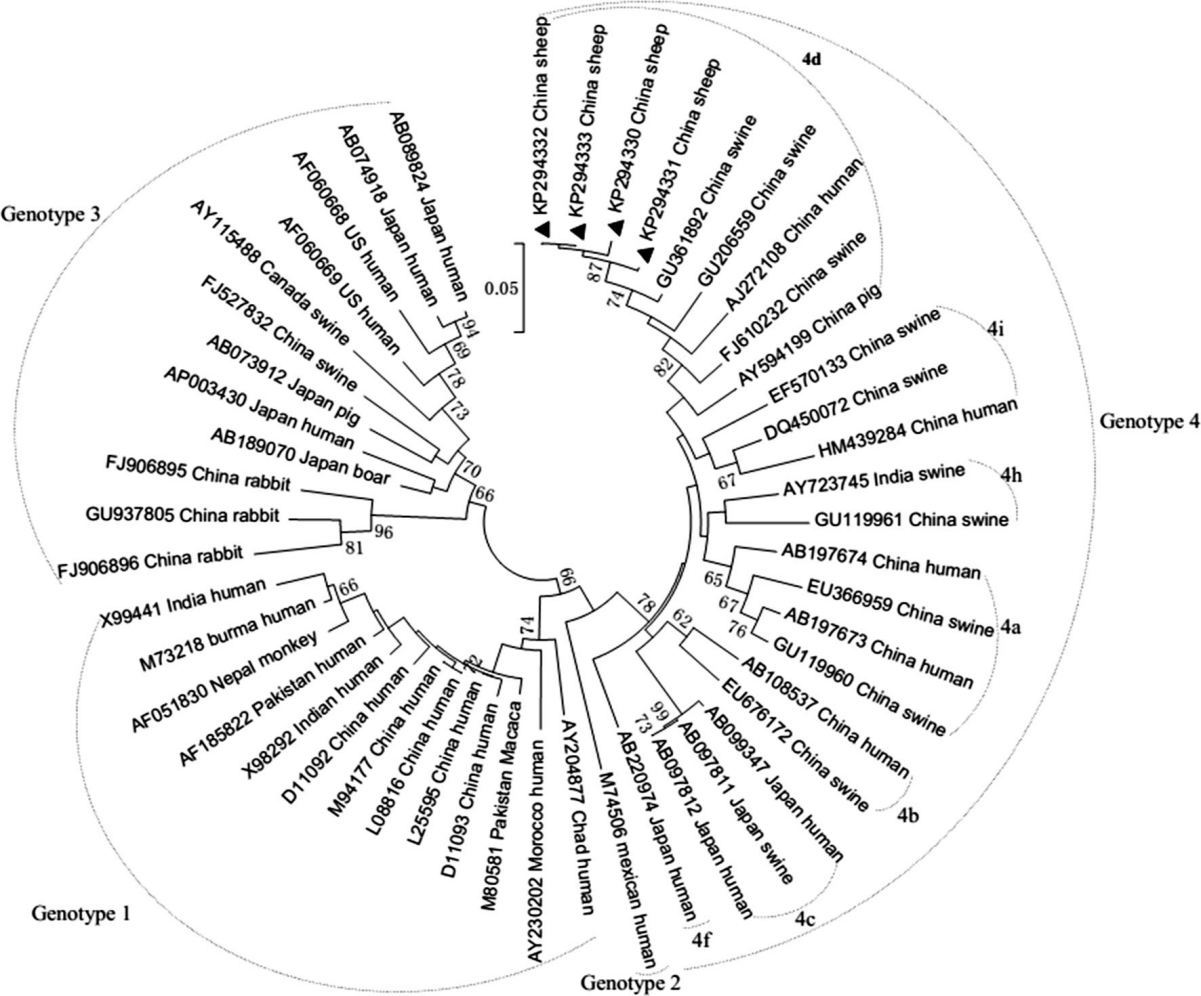
Phylogenetic tree based on a 186-bp nucleotide sequences of HEV-ORF2. The tree was constructed by the neighbour-joining method using 46 animal and human HEV reference strains. Strains characterised in this study were indicated with symbol *filled triangle*. The internal node numbers indicated the *bootstrap values* as a percentage obtained from 1000 replications. The *Arabic numbers* and the *English letters* outside of the *dashed curves* indicate the genotype and subtype. The *bar* on the tree shows the scale of 0.05 % nucleotide substitution per site. GenBank accession numbers, country and species of origin are indicated for each isolate represented on the tree

ORF2 fragments (186 bp) amplified from the sheep HEV genome were compared with the corresponding sequences of other human and swine HEV strains deposited in GenBank. Sequence analysis indicated that these four sheep HEV strains designated as XJNJSLhev25, XJNJSLhev27, XJNJSLhev28 and XJNJSLhev30 (GenBank accession numbers KP294330- KP294333) shared 97.8–100 % nucleotide homology with each other and had identities of 81.6–85.3 %, 84.2–85.3 %, 82.1–85.3 % and 84.7–97.9 % with the corresponding regions of reference HEV genotypes 1, 2, 3 and 4, respectively (Table 2). A high degree of identity was observed between the strains found in the current study and those described in a previous report, including hb-3 (GU361892.1), bjsw1 (GU206559.1), T1 (AJ272108.1), swCH189 (FJ610232.1) and swCH25 (AY594199.1). Further phylogenetic analysis based on alignment of partial ORF2 sequences revealed that the four strains in the current study formed a single lineage and were on the same branch as hb-3, bjsw1, T1, swCH189 and swCH25, all of which belong to genotype 4, subtype 4d [66] (Fig. 2). This finding suggested a common origin for the currently circulating HEV strains in this region.

**Table 2:** Pairwise sequence identities between the four sheep liver HEV sequences and corresponding sequences of reference strains of the four genotypes (%)

Sequences^a^	1(13)	2(1)	3(12)	4(20)	Sheep sequences
Sheep sequences	81.6–85.3	84.2–85.3	82.1–85.3	84.7–97.9	97.8–100.0
4(20)	80.5–88.4	83.7–86.8	78.9–86.8	86.3–100.0	
3(12)	80.5–91.1	80.0–86.8	80.5–100.0		
2(1)	83.7–87.9	100.0			
1(13)	88.9–100.0				

^a^Numbers out of parentheses represent genotypes of reference sequences. Numbers in parentheses represent number of reference sequences

## Discussion

HEV is a cause for public health concern in many developing countries where sanitation conditions are poor and waterborne epidemics are frequent. Both epidemic and sporadic forms of HEV infections exist in developing and under-developed countries. Nevertheless, accumulating evidence indicates that HEV-associated hepatitis can also occur in individuals living in industrialised countries and that zoonotic sources may be responsible for such cases [4–6, 21, 61]. Swine are believed to be the principal reservoir of HEV, but other animals, including cattle, dogs and rodents, have also been reported to be highly seropositive for anti-HEV IgG [67]. Given the high infection rates and expanded HEV host range, increasing attention has been focused on the zoonotic nature of HEV and the close association of particular animal species with humans. In fact, numerous cases of human HEV sharing high genomic sequence identity with virus isolates from local animals have recently been reported [7, 68, 69]. All of these findings strongly support a zoonotic transmission, which also explains the cause of autochthonous HEV infections in developed countries. Furthermore, numerous examples of human infections via consumption of contaminated raw meat or other animal products have surfaced in recent years. In Germany, autochthonous HEV infection was found to be associated with the consumption of offal and wild boar meat [70]. Similar events have occurred in Japan, France and the USA [7, 68, 69]. These events alert us to the necessity for constant surveillance of HEV transmission routes in order to prevent foodborne epidemics.

In China, endemic hepatitis E can be traced back to historical outbreaks in the 1980s in the Xinjiang Autonomous Region, which has had the highest prevalence of HEV infections worldwide. Several human and animal viral genomes have been isolated, and phylogenetic analysis indicated that dominant HEV genotypes in this area have converted from genotype 1 to genotype 4 [71, 72]. Interestingly, human HEV has been found mainly in Uighurs, prompting a series of questions such as whether this predilection is the result of a genetic predisposition or of agricultural, social or food habits, whether there are underlying co-morbidities, and what the principal infective agents are. Here we focused particularly on the food habits of the local inhabitants, of whom the majority are Uighurs. For Uighurs, sheep meat is common in their diet. They consume grilled meat, but also sheep liver and other inner organs, and this was one of the major reasons why we focused our study on sheep HEV. Our results indicated a high HEV antibody prevalence in sheep herds in these areas [64]. Interestingly, we found that by comparison with our seroprevalence study performed previously in the same area, the seroprevalence of anti-HEV antibody in sheep was now even higher, indicating that sheep HEV is still circulating in this region. Furthermore, two different age groups of sheep showed significant differences in seropositivity for anti-HEV antibody, with seroprevalence increasing with age, possibly as a consequence of repeated contact with the virus. In sheep less than 1 year of age, the seroprevalence rate was 10.7 %, and in sheep 2 years or older, it was 45.7 %. None of the 500 sheep serum samples were positive for HEV RNA, and approximately 5.3 % (4 of 75) of raw sheep livers were HEV-positive. The fact that the seroprevalence rate was higher than the percentage of HEV-positive livers suggests that the period of viraemia is relatively short. One might argue that the low frequency of virus isolation from livers may be due to variations in virus titers in different parts of this large organ. Indeed, samples taken from 5 to 10 locations of the same liver showed differences in virus positivity, but samples from many other organs including muscle and several lymph nodes as well as excretions such as bile, intestinal contents and faeces were consistently negative. Hence, the liver still gave us the best chance of finding evidence for the presence of virus.

Another interesting aspect of our study was the fact that sheep and slaughterhouse workers showed antibodies to the same antigen. We reached this conclusion because we used the same ELISA kit capable of detecting antibodies to highly purified ORF2 capsid protein of human HEV for both sheep and human sera. This finding suggested that the immune systems of both sheep and humans recognise an overlapping set of immunogenic HEV proteins.

The phylogenetic tree (Fig. 2) revealed that the strains identified in this study were most closely related to a human HEV strain (T1, AJ272108.1) and four swine HEV strains (hb-3, GU361892.1; bjsw1, GU206559.1; swCH189, FJ610232.1; swCH25, AY594199.1). The strain swCH25 is a swine HEV strain isolated in the area of Xinjiang, and the human HEV strain and the remaining three swine HEV strains were identified in different areas. However, all of them belong to genotype 4, subtype 4d. Further epidemiological surveys should therefore be carried out to confirm whether subtype 4d is the predominant strain circulating in this region of China.

## Conclusion

Our study showed for the first time that HEV RNA can be detected in sheep liver samples. Hence, our results provide strong evidence that sheep can be, and are, infected with HEV and that sheep products, in particular liver and perhaps other inner organs, can be a source of food born infections in humans. Moreover, it appears that slaughterhouse workers with extensive contact with sheep show a high rate of HEV seroconversion. Our results underscore the need for further studies to clarify epidemiological factors such as the distinct pattern of sheep HEV distribution and the extent of cross-species transmission.
